# Terrestrial evidence for volcanogenic sulfate-driven cooling event ~30 kyr before the Cretaceous–Paleogene mass extinction

**DOI:** 10.1126/sciadv.ado5478

**Published:** 2024-12-18

**Authors:** Lauren K. O’Connor, Rhodri M. Jerrett, Gregory D. Price, Tyler R. Lyson, Sabine K. Lengger, Francien Peterse, Bart E. van Dongen

**Affiliations:** ^1^ Department of Earth and Environmental Sciences, University of Manchester, Manchester, UK.; ^2^ Department of Earth Sciences, Faculty of Geosciences, Utrecht University, Utrecht, Netherlands.; ^3^ School of Geography, Earth and Environmental Sciences, Plymouth University, Plymouth, UK.; ^4^ Department of Earth Sciences, Denver Museum of Nature & Science, Denver, CO, USA.; ^5^ Sensor Systems Division, Silicon Austria Labs, Villach, Austria.

## Abstract

Alongside the Chicxulub meteorite impact, Deccan volcanism is considered a primary trigger for the Cretaceous–Paleogene (K–Pg) mass extinction. Models suggest that volcanic outgassing of carbon and sulfur—potent environmental stressors—drove global temperature change, but the relative timing, duration, and magnitude of such change remains uncertain. Here, we use the organic paleothermometer MBT′_5me_ and the carbon-isotope composition of two K–Pg-spanning lignites from the western Unites States, to test models of volcanogenic air temperature change in the ~100 kyr before the mass extinction. Our records show long-term warming of ~3°C, probably driven by Deccan CO_2_ emissions, and reveal a transient (<10 kyr) ~5°C cooling event, coinciding with the peak of the Poladpur “pulse” of Deccan eruption ~30 kyr before the K–Pg boundary. This cooling was likely caused by the aerosolization of volcanogenic sulfur. Temperatures returned to pre-event values before the mass extinction, suggesting that, from the terrestrial perspective, volcanogenic climate change was not the primary cause of K–Pg extinction.

## Teaser

Terrestrial proxy evidence for sulfur-induced cooling event linked to Deccan Trap eruptions 30 ka before K–Pg boundary.

## INTRODUCTION

The Cretaceous–Paleogene (K–Pg) boundary [~66 million years ago (Myr)] represents the most recent mass extinction event; an estimated 75% of all species were extinguished (*1*), including all nonavian dinosaurs. This event changed the trajectory of the evolutionary tree of life (*2*, *3*) and resulted in a complete rebuilding of ecosystems from dinosaur- to mammal-dominated communities. The Chicxulub meteorite impact [Mexico (*4*–*6*)] and eruption of the Deccan Traps [India (*7*–*12*)] have emerged as the primary—but fiercely contested—trigger mechanisms for the mass extinction and global climate change. Models of the climate response to the meteorite impact include an “impact winter” lasting months to millennia due to atmospheric loading of dust, soot, and sulfate aerosols (*13*–*17*), and longer-term warming caused by CO_2_ released by wildfires and/or impact-volatilized carbonates (*18*). Two principal climate models are associated with Deccan volcanism: first, global warming, caused by eruption-, venting-, and contact-metamorphism-derived CO_2_ (*19*) and sustained over thousands to hundreds of thousands of years [e.g., (*20*)]; and second, global cooling driven by the conversion of SO_2_ into sulfate aerosols, but lasting only for the duration of the eruption (*21*–*23*).

Recent high-precision radiometric dating has established synchronicity between meteorite impact and extinction (*24*, *25*) and has shown that the most major phase of Deccan volcanism—the Poladpur “pulse” [sensu Schoene *et al.* (*26*)]—erupted from 66.10 to 66.00 Myr, peaking 30 thousand years ago (kyr) before the K–Pg boundary (*26*, *27*). These data do not exclude Deccan volcanism as a contributing or primary cause of extinction, but rather provide a high-precision geochronology of events around the K–Pg boundary against which competing hypotheses of climate change can be tested using age-constrained proxy reconstructions.

**Fig. 1.**
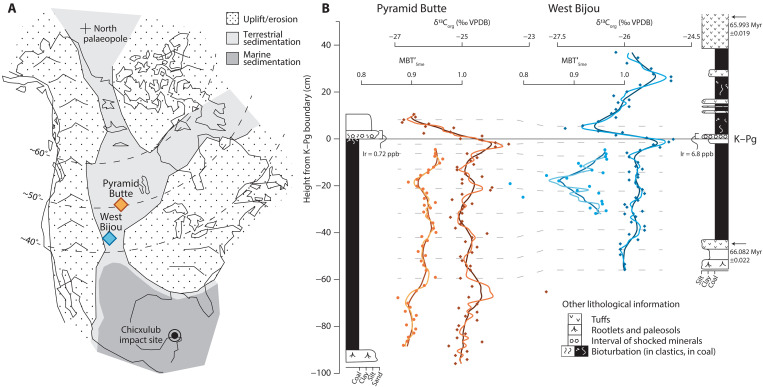
Paleogeographic context of North America highlighting the study locations, alongside physical stratigraphy, MBT′_5me_, and bulk-organic δ^13^C records for West Bijou (blue) and Pyramid Butte (orange). (**A** and **B**) Medium and dark solid lines through the records show three- and five-point moving averages, respectively. Dashed lines show the correlation between sites. The stratigraphic levels of tuffs are shown, with their radiometric dates (*25*). The solid gray line denotes the position of the K–Pg boundary—the primary tiepoint—as defined by the presence of shocked minerals, iridium anomalies, and palynological extinctions at Pyramid Butte (*32*, *66*) and West Bijou (*33*, *65*). Note that data filtering (Supplementary Materials) precludes the generation of MBT′_5me_ records for the entirety of the lignites, and a short gap exists immediately below the K–Pg boundary.

Here, we reconstruct mean annual air temperatures (MAATs) at a millennial resolution leading up to the K–Pg boundary based on branched glycerol dialkyl glycerol tetraethers (brGDGTs) in fossil peats (lignites) from two mid-paleolatitude sites in the Western Interior of the United States (Pyramid Butte, North Dakota, and West Bijou, Colorado; Fig. 1). BrGDGTs are membrane lipids of bacteria living in soils and peats that adjust the number of methyl branches attached to the alkyl backbone to changes in temperature. The degree of methylation is quantified in the Methylation index of 5-methyl Branched glycerol dialkyl glycerol Tetraethers [MBT′_5me_ index (*28*, *29*)] and can be translated into MAAT using a peat-specific transfer function (*30*). MBT′_5me_ is an established proxy that has been successfully used for deep-time paleotemperature reconstruction (*31*), though confounding factors may influence the temperature signal reflected by the brGDGT distributions stored in sedimentary archives. Hence, brGDGT distributions need to be carefully assessed before their interpretation as a temperature signal. We have, therefore, developed a framework that allowed us to identify samples with potential non-thermal influences on brGDGT distributions (see the Supplementary Materials).

Although the peats at both sites accumulated in discrete depocenters of the Western Interior Basin, the K–Pg boundary is clearly identifiable at each by the presence of diagnostic meteorite-impact indicators (shocked minerals and an iridium anomaly) and coincident palynomorph extinction (Fig. 1B) (*32*, *33*). This datum forms the principal basis of correlation between our sites. In addition, a succession of primary volcanic tuffs interbedded with the lignites at West Bijou bracket the K–Pg boundary (*25*). Critically, these tuffs were dated (*25*) using the same method and calibration as Schoene *et al.*’s (*26*) study of Deccan eruptions [compare Sprain *et al.* (*34*)] and, for this reason, we focus on these dates. The lower tuff, KJ08157 (~10 cm thick), the top of which occurs 43 cm below the palynological K–Pg boundary and defines the base of the lignite seam, yielded an age of 66.082 ± 0.022 Myr (*25*). The base of tuff KJ0475 (~13 cm thick) occurs 52 cm above the palynological K–Pg boundary and yielded an age of 65.993 ± 0.019 Myr (*25*). Linear interpolation (of peat accumulation rates and peat-to-lignite compaction ratios) between these two dates implies 1 m of lignite = 97 kyr ± 45 kyr. Last, the bulk-organic carbon-isotope (δ^13^C) records at both sites provide a secondary correlation framework for our study. Our high-resolution geochronological model suggests that our filtered MAAT record spans the last ~50 kyr and ~100 kyr of the Cretaceous at West Bijou and Pyramid Butte, respectively. This interval encompasses the main eruptive phase of Deccan volcanism—the Poladpur phase (*26*).

We compare these reconstructions with bulk-organic carbon-isotope (δ^13^C) records generated from the same samples to determine relationships between climate and carbon cycling at this time. Our data, therefore, directly test hypotheses of terrestrial climate change driven by Deccan volcanism, particularly eruption of the Poladpur Formation, which overlaps temporally with our MAAT record (*26*).

## RESULTS

**Fig. 2.**
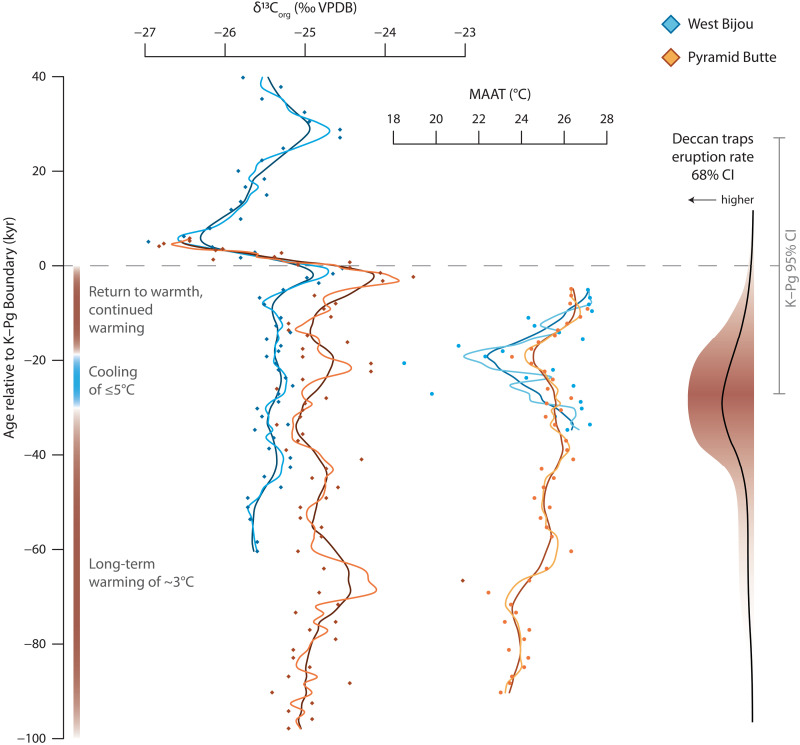
Atmospheric-CO_2_ δ^13^C and mean annual air temperatures (MAAT) plotted against time (kyr) relative to the K–Pg boundary. The medium and dark solid lines through the records show three- and five-point moving averages, respectively. The 95% confidence interval (CI) of the K–Pg boundary (dashed line), as determined by Schoene *et al.* (*26*), is indicated in gray. The probabilistic volumetric eruption rate for the Deccan Traps (*26*) is shown on the right (red shading shows the 68% CI and the black line shows the mean value); the greatest volume was likely erupted between ~50 and 10 kyr before the K–Pg boundary, with the peak around 30 kyr before.

We present our reconstructed MAATs using the MBT′_5me_ proxy calibration developed for peats [calibration error ±4.7°C (*30*)] and the abovementioned age model. Over the last ~100 kyr of the Cretaceous, MAATs ranged from ~24° to 27°C at Pyramid Butte and from ~21° to 27°C at West Bijou (Fig. 2). There is remarkable synchroneity between the two temperature records where they overlap stratigraphically. We highlight three distinct intervals (Fig. 2): (i) 100 to 30 kyr before the K–Pg boundary MAATs increased from 23° to 26°C, with a stepwise increase 70 kyr before the boundary (this trend is derived from the Pyramid Butte record); (ii) from 30 to 20 kyr before the K–Pg boundary, MAATs declined to a minimum of 23°C at Pyramid Butte and from 27° to 21°C at West Bijou; (iii) during the last ~20 kyr of the Cretaceous, MAATs at both sites returned to pre-event values and then stabilized at 27°C. Thus, both sites display a markedly symmetrical cooling event, with a magnitude of change of 2° to 5°C, beginning 30 kyr before the K–Pg boundary and lasting no more than 10 kyr. This trend is superimposed on a longer-term (at least 100 kyr) latest Maastrichtian warming of 3°C.

The δ^13^C values range from −26.8 to −23.6‰ and from −27.3 to −24.9‰ at Pyramid Butte and West Bijou, respectively. In the latest Cretaceous part of the record, the values are remarkably consistent, deviating little from a mean around −26‰ at West Bijou and −25‰ at Pyramid Butte (Fig. 2), until the distinct negative excursion that characterizes the K–Pg boundary worldwide (*35*–*41*).

## DISCUSSION

Our two sites show remarkable similarity in absolute MAATs and temporal trends, despite being located 750 km apart, providing confidence that the individual sites are recording a regional climatic signal, and bolstering support for the reliability of the MBT′_5me_ proxy [e.g., (*42*)]. Furthermore, the absolute MAAT values are similar to those reconstructed for the latest Maastrichtian from lignites in nearby southern Saskatchewan [Canada; 20° to 25°C (*31*)], and clumped-isotope paleothermometry on terrestrial mollusks in Montana (USA; 23° to 30°C) (*43*). Likewise, the δ^13^C records at both our sites are notably similar to one another (Figs. 1 and 2) and to other terrestrial records (*40*). Our record therefore reflects a global signal of atmospheric-CO_2_ δ^13^C (*38*, *44*).

There are few single-proxy paleotemperature studies at the resolution of this study against which even the longer-term trend in our study—MAAT rise of 3°C over the last 100 kyr of the Cretaceous—can be compared. However, warming in the lead-up to the boundary is captured in both marine-carbonate δ^18^O (*39*) and terrestrial brGDGT (*31*) records. Certainly, this latest-Maastrichtian warming trend in our data postdates the Late Maastrichtian Warming Event (LMWE) of ~5°C dated to ~300 to 200 kyr before the K–Pg boundary (*39*, *41*, *45*).

The cause of the 3°C warming in our record can circumstantially be linked to Deccan volcanism. U–Pb radiometric dating reveals that the Deccan Traps were erupted as four discrete high-volume events separated by relative volcanic quiescence (*26*). These include an event 300 to 150 kyr before the K–Pg boundary (*39*, *44*), and another—the Poladpur pulse—centered on the last 100 kyr of the Cretaceous (*26*). These events, or pulses, in fact comprised multiple eruptions that individually lasted decades to centuries (*26*), with peak eruptive volumes of 5 to 10 km^3^/year occurring 30 kyr before the K–Pg boundary (*26*). [We note, though, that ^40^Ar/^39^Ar dating undertaken by Sprain *et al.* (*34*) suggests a more continuous eruption style for Deccan volcanism.] Both the LMWE and the later warming trend observed in our data correspond to the onset and duration of these two eruptive events: the Kalsubai/Lonavala and Poladpur, respectively. Gilabert *et al.* (*46*) have previously attributed the LMWE to increased CO_2_ associated with emplacement of the Kalsubai/Lonavala subgroup combined with background orbital forcing. The length of our combined record (~100 kyr) does not permit the testing of orbital cyclicity in a statistically significant way. However, the recognition of two distinct warming episodes—the LMWE and the later warming captured in our record—is inconsistent with the notion of quasicontinuous eruption (and CO_2_ release) [e.g., (*34*)].

Models based on the understood volumes and rates of total Deccan lava eruption, and assumptions about associated CO_2_ emissions imply that they resulted in net global warming of less than 1°C (*47*) to 4°C (*19*, *20*, *48*). Recent geochemical analyses of accompanying intruded (i.e., unerupted) magmas increase total CO_2_ emission estimates and imply that up to 6°C warming may have been possible (*49*) but diminish the temporal connection between lava eruptions and the tempo and rates of CO_2_ outgassing.

The time interval reconstructed in this study (~100 kyr) represents a fraction of the total Deccan eruptive time, and the Poladpur Formation represents less than a quarter of the volume of the Deccan Traps. Therefore, the ~3°C of warming we observe in our records appears possibly high compared to model outputs based on the entire Deccan Traps. We do not consider the paleolatitudes of our two sites (45°N and 51°N for West Bijou and Pyramid Butte, respectively) to be sufficiently high to invoke polar amplification as an explanation for the relatively large rise in MAAT that we observe. As an explanation, Hernandez Nava *et al.* (*49*) found that late Deccan magmas became depleted in CO_2_, meaning that the earlier phases of outgassing were more efficient in driving global warming. Furthermore, combined biological and mineral reaction sequestration rates could not fully offset the volumes of CO_2_ injected into the atmosphere at the timescales between individual Deccan events (*20*, *48*), resulting in diminishing greenhouse effects of each new mole of CO_2_ emitted (*50*). The 5°C of LMWE warming that has been ascribed to the first Deccan eruptive event 300 to 200 kyr before the K–Pg boundary is greater than the 3°C observed in our record, consistent with this hypothesis (*45*). We propose that subsequent eruptive phases would show decreasing effects on global temperatures.

Transfer of CO_2_ or CH_4_ to the atmospheric reservoir may have also occurred through the metamorphism and/or combustion of hydrocarbons associated with the emplacement of the Deccan Traps [e.g., (*51*–*53*)]. Further, CO_2_ may have been released from the terrestrial and marine biogenic reservoirs, caused by a latest Maastrichtian ecological stress and reduction in biomass (*54*, *55*). These organic, isotopically light [−29‰ (*56*)] carbon sources would have resulted in a distinct imprint on our atmospheric δ^13^C record in the form of a negative excursion. The only notable negative excursion in our data corresponds to the K–Pg boundary itself (Figs. 1 and 2) and postdates our temperature record. There is no correspondence between our MAAT and atmospheric δ^13^C record (*r*^2^ of regression = 0.005), so we preclude a major organic source of CO_2_ as the driver of this longer-term warming phase. This finding supports the argument that organic carbon played only a minor role in the earlier Deccan-associated LMWE (*45*), despite assertions that warming in the absence of a negative δ^13^C excursion does not preclude protracted addition of isotopically light carbon (*53*). By comparison, volcanogenic carbon has a comparatively heavy δ^13^C signature [−5‰ (*56*)] requiring much greater carbon emissions to affect change in atmospheric δ^13^C (*56*), and to be detectable in the sedimentary geochemical record (*57*, *58*). The ~3°C of warming observed in our records is therefore consistent with the greenhouse effects of CO_2_ emissions associated with the onset of the Poladpur pulse.

The prominent, ~10 kyr duration, 2° to 5°C cooling event observed in both of our records has not previously been recognized in terrestrial paleotemperature reconstructions. Investigations of any such cooling linked with Deccan volcanism have—until now—been limited by the paucity of well-dated high-resolution temperature records [e.g., (*43*)]. The well-dated marine δ^18^O record of Keller *et al.* (*12*) does recognizes a cooling event ~45 to 25 kyr before the K–Pg boundary (*12*), which overlaps with our event within error of the respective age models, though the authors attributed the cooling instead to a cessation of volcanogenic CO_2_ release. Nonetheless, several lines of evidence imply that our record is the first, to our knowledge, to test and support recent models of global cooling driven by the conversion of erupted SO_2_ into sulfate aerosols for short periods [e.g., (*21*–*23*, *59*, *60*)].

First, the timing of the cooling event, beginning ~30 kyr before the K–Pg boundary, coincides with peak rates of Poladpur Formation lava emplacement (*26*), using the same U–Pb dating methods and calibration technique as the tuff ages at West Bijou (*25*) (Fig. 2). Sulfur outgassing occurs at, or close to, the extrusive surface (*49*); thus—unlike CO_2_ emissions associated with a combination of intrusive and extrusive processes—SO_2_ emissions must be shown to be contemporaneous with the Deccan eruptive phases. Studies of stratigraphic mercury (Hg) in combination with high-precision ^40^Ar/^39^Ar tuff dates from lignite-bearing K–Pg successions in the Williston Basin (Montana) have, additionally, revealed elevated concentrations ~30 kyr pre-boundary (*61*). Fendley *et al.* (*61*) attributed the Hg spike to a pulse of Deccan volcanism, which they determined from overall Hg concentrations to have lasted on the order of several centuries.

Individual Deccan lavas were erupted over decades to centuries (*26*, *62*). Volcanogenic sulfur emissions are modeled to have driven cooling of 5° to 8°C, sustained for the duration of individual eruptions, after which the aerosols would be removed within ~50 years as acid rain (*21*). The cooling event observed at both West Bijou (5°C) and in particular Pyramid Butte (2°C) is of a slightly lesser magnitude than modeled by Schmidt *et al.* (*21*), and at least an order of magnitude longer in duration. These discrepancies can both be explained by (i) the higher heat capacity of water in the saturated peat compared with air, introducing a dampened signal in the brGDGT record, or (ii) the effect of mixing of organic material in the peat profile, as younger plants grow roots into and remobilize older peat. As such, our data imply that there is a limit to the temporal resolution of lignite records such as these. The notion that the climatic signal has been overprinted by these processes is supported by the difference in reported magnitude of the negative temperature excursion between the two sites; it is unlikely that localities separated by 750 km and showing such similar trends and absolute values of MAAT over the longer term should experience such a substantial difference in SO_2_ aerosol-induced cooling. Although the markedly symmetrical nature of the cooling and then warming could be interpreted as the onset, acme, and waning phases of volcanic activity, we posit that it reflects the smoothed expression of a more discrete, shorter-duration and higher-magnitude cooling event. We consider the expression of the transient cooling event in our data to represent an absolute minimum in terms of magnitude, a maximum in terms of duration, and consistent with models of the Deccan-sourced SO_2_-induced cooling (*21*–*23*).

The cooling event does not coincide with any excursions in our atmospheric δ^13^C record (Fig. 2), as would be expected if it was caused by orbital forcing and/or biogenic sequestration of CO_2_ and a reduction in greenhouse effect. Consequently, as with the longer-term warming observed in our record, interpretations of cooling as generated by volcanism does not necessitate a perturbation to the carbon-isotope composition of atmospheric CO_2_.

Overall, our data show a longer-term warming signal of ~3°C over the last 100 kyr of the Cretaceous, on which a transient cooling event of 2° to 5°C is superimposed, centered on ~30 kyr before the K–Pg boundary. To a degree, our data therefore support both models of climate change induced by Deccan volcanism, that is, longer-term (>100 kyr) warming caused by Deccan volcanogenic and magmatic outgassing of CO_2_ (*19*, *49*), and short-term (<10 kyr), high-magnitude (5°C) cooling caused by the conversion of volcanogenic SO_2_ into sulfate aerosols [e.g., (*21*)]. This study is the first, to our knowledge, to test and support the latter model, the climate response of which has been asserted as being too short in duration to be detectable in the stratigraphic record (*26*).

Peat accumulation is relatively steady and at longer timescales, fast, compared to clastic floodplain sedimentation (*63*), and this study emphasizes the utility of lignite and coal records as sensitive archives of deep-time terrestrial climate change. Our record highlights the varying tempo of Deccan volcanism–induced climate change directly tied to the different timescales of carbon and sulfur cycling. The longer-term, CO_2_-induced warming of the latest Maastrichtian can now be separated into two discrete events: the LMWE and that of our new record, both coinciding with pulses of Deccan eruption. The latter was punctuated by a brief cooling event linked to sulfur cycling, and similar events may have occurred during other eruptive events but have not yet been resolved in paleoclimate records. Further study of, for example, longer lignite records spanning the K–Pg boundary [e.g., (*64*)] with high-precision chronological constraint will test the notion that each of the main Deccan eruptive phases will be accompanied by concomitant short-duration cooling.

To what degree were these climatic phenomena implicated in the mass extinction at the K–Pg boundary? The 5°C cooling and subsequent return to pre-event warmth, which we attribute to the effect of aerosolized SO_2_ emissions by the Poladpur eruption, predates the palynological extinction at the K–Pg boundary at both West Bijou (*33*, *65*) and Pyramid Butte (*32*, *66*) by several millennia. Despite a relatively coarse sampling density (12 samples for the whole of the lignite at Pyramid Butte, as opposed to the *n* = 51 data points for this section in our record), Nichols and Johnson (*66*) show that of the 17 angiosperm taxa that predate the transient cooling episode, 15 continue to the K–Pg boundary or into the Paleocene. Therefore, while this rapid climate change associated with volcanogenic aerosolized SO_2_ emissions may have induced ecological stress, from a palynological perspective, it was not the primary cause of the extinction at the K–Pg boundary.

## MATERIALS AND METHODS

The lignite seams at Pyramid Butte (46°25′03″N 103°58′33″W) and West Bijou (39°34′14″N 104°18′09″W) were sampled contiguously, and samples were freeze dried and powdered before geochemical analysis. Samples were solvent extracted using a MARS6 microwave extraction system, and the total lipid extracts were separated into polar and apolar fractions using aluminum oxide column chromatography. The polar fractions (containing the GDGTs) were filtered (0.45 μm polytetrafluoroethylene, PTFE) using hexane:isopropanol (99:1, v/v) before analysis by high-performance liquid chromatography/atmospheric pressure chemical ionization–mass spectrometry (HPLC/APCI-MS) on a TSQ Quantum Access Orbitrap HPLC-MS [University of Plymouth (*31*)]. Samples were screened for nonthermal influences on GDGTs using several GDGT indices: the Branched and Isoprenoid Tetraether index (*67*), relative abundance of 6-methyl versus 5-methyl brGDGTs (*68*), degree of cyclization (*68*), and community index (*69*). After the exclusion of samples biased by nonthermal influences (Supplementary Materials), the MBT′_5me_ index was calculated following De Jonge *et al.* (*29*). The peat-specific calibration [MAAT_peat_ (*30*)] was used to convert MBT′_5me_ values into MAATs. Bulk-organic δ^13^C analyses were conducted at Plymouth University using an Isoprime mass spectrometer connected to an Isoprime Microcube elemental analyzer.

## Supplementary Materials

### The PDF file includes:

Supplementary Text Figs. S1 and S2 Legend for data S1 References

## References

[1] N. MacLeod, P. F. Rawson, P. L. Forey, F. T. Banner, M. K. Boudagher-Fadel, P. R. Bown, J. A. Burnett, P. Chambers, S. Culver, S. E. Evans, C. Jeffery, M. A. Kaminski, A. R. Lord, A. C. Milner, A. R. Milner, N. Morris, E. Owen, B. R. Rosen, A. B. Smith, P. D. Taylor, E. Urquhart, J. R. Young, The Cretaceous-Tertiary biotic transition. J. Geol. Soc. 154, 265–292 (1997).

[2] M. A. O’Leary, J. I. Bloch, J. J. Flynn, T. J. Gaudin, A. Giallombardo, N. P. Giannini, S. L. Goldberg, B. P. Kraatz, Z.-X. Luo, J. Meng, X. Ni, M. J. Novacek, F. A. Perini, Z. S. Randall, G. W. Rougier, E. J. Sargis, M. T. Silcox, N. B. Simmons, M. Spaulding, P. M. Velazco, M. Weksler, J. R. Wible, A. L. Cirranello, The placental mammal ancestor and the post–K–Pg radiation of placentals. Science 339, 662–667 (2013). 23393258 10.1126/science.1229237

[3] P. Hull, Life in the aftermath of mass extinctions. Curr. Biol. 25, R941–R952 (2015). 26439357 10.1016/j.cub.2015.08.053

[4] L. W. Alvarez, W. Alvarez, F. Asro, H. V. Michel, Extraterrestrial cause for the Cretaceous-Tertiary extinction. Science 208, 1095–1108 (1980). 17783054 10.1126/science.208.4448.1095

[5] A. R. Hildebrand, G. T. Penfield, D. A. Kring, M. Pilkington, A. Camargo, S. B. Jacobsen, W. V. Boynton, Chicxulub crater—A possible Cretaceous-Tertiary boundary impact crater on the Yucatan Peninsula, Mexico. Geology 19, 867–871 (1991).

[6] P. Schulte, L. Alegret, I. Arenillas, J. A. Arz, P. J. Barton, P. R. Bown, T. J. Bralower, G. L. Christeson, P. Claeys, C. S. Cockell, G. S. Collins, The Chicxulub asteroid impact and mass extinction at the Cretaceous-Paleogene boundary. Science 327, 1214–1218 (2010). 20203042 10.1126/science.1177265

[7] V. Courtillot, J. Besse, D. Vandamme, R. Montigny, J. J. Jaeger, H. Cappetta, Deccan flood basalt at the Cretaceous/Tertiary boundary? Earth Planet. Sci. Lett. 80, 361–374 (1986).

[8] R. A. Duncan, D. G. Pyle, Rapid eruption of the Deccan flood basalts at the Cretaceous/Tertiary boundary. Nature 333, 841–843 (1988).

[9] V. Courtillot, F. Fluteau, Cretaceous extinctions: The volcanic hypothesis. Science 328, 973–974 (2010). 10.1126/science.328.5981.973-b 20489003

[10] B. Gertsch, G. Keller, T. Adatte, R. Garg, V. Prasad, Z. Berner, D. Fleitmann, Environmental effects of Deccan volcanism across the Cretaceous-Tertiary transition in Meghalaya, India. Earth Planet. Sci. Lett. 310, 272–285 (2011).

[11] E. Font, T. Adatte, A. N. Sial, L. D. de Lacerda, G. Keller, J. Punekar, Mercury anomaly, Deccan volcanism, and the end-Cretaceous mass extinction. Geology 44, 171–174 (2016).

[12] G. Keller, P. Mateo, J. Monkenbusch, N. Thibault, J. Punekar, J. E. Spangenberg, S. Abramovich, S. Ashckenazi-Polivoda, B. Schoene, M. P. Eddy, K. M. Samperton, Mercury linked to Deccan Traps volcanism, climate change and the end-Cretaceous mass extinction. Global Planet. Change 194, 103312 (2020).

[13] K. O. Pope, K. H. Baines, A. C. Ocampo, B. A. Ivanov, Impact winter and the Cretaceous/Tertiary extinctions: Results of a Chicxulub asteroid impact model. Earth Planet. Sci. Lett. 128, 719–725 (1994). 11539442 10.1016/0012-821x(94)90186-4

[14] C. G. Bardeen, R. R. Garcia, O. B. Toon, A. J. Conley, On transient climate change at the Cretaceous-Paleogene boundary due to atmospheric soot injections. Proc. Natl. Acad. Sci. U.S.A. 114, E7415–E7424 (2017). 28827324 10.1073/pnas.1708980114 PMC5594694

[15] J. Brugger, G. Feulner, S. Petri, Baby, it’s cold outside: Climate model simulations of the effects of the asteroid impact at the end of the Cretaceous. Geophys. Res. Lett. 44, 419–427 (2017).

[16] C. B. Senel, P. Kaskes, O. Temel, J. Vellekoop, S. Goderis, R. DePalma, M. A. Prins, P. Claeys, Ö. Karatekin, Chicxulub impact winter sustained by fine silicate dust. Nat. Geosci. 16, 1033–1040 (2013).

[17] C. B. Officer, A. Hallam, C. L. Drake, J. D. Devine, Late Cretaceous and paroxysmal Cretaceous/Tertiary extinctions. Nature 326, 143–149 (1987).

[18] J. D. O’Keefe, T. J. Ahrens, Impact production of CO_2_ by the Cretaceous/Tertiary extinction bolide and the resultant heating of the Earth. Nature 338, 247–249 (1989).

[19] S. Self, A. Schmidt, T. A. Mather, Emplacement characteristics, time scales, and volcanic gas release rates of continental flood basalt eruptions on Earth. Geol. Soc. Am. Spec. Pap. 505, 319–337 (2014).

[20] C. Dessert, B. Dupre, L. M. Francois, J. Schott, J. Gaillardet, G. Chakrapani, S. Bajpai, Erosion of Deccan Traps determined by river geochemistry: Impact on the global climate and the Sr-87/Sr-86 ratio of seawater. Earth Planet. Sci. Lett. 188, 459–474 (2001).

[21] A. Schmidt, R. A. Skeffington, T. Thordarson, S. Self, P. M. Forster, A. Rap, A. Ridgwell, D. Fowler, M. Wilson, G. W. Mann, P. B. Wignall, Selective environmental stress from sulfur emitted by continental flood basalt eruptions. Nat. Geosci. 9, 77–82 (2016).

[22] S. Callegaro, D. R. Baker, P. R. Renne, L. Melluso, K. Geraki, M. J. Whitehouse, A. De Min, A. Marzoli, Recurring volcanic winters during the latest Cretaceous: Sulfur and fluorine budgets of Deccan Traps lavas. Sci. Adv. 9, eadg8284 (2023). 37792933 10.1126/sciadv.adg8284 PMC10550224

[23] A. A. Cox, C. B. Keller, A Bayesian inversion for emissions and export productivity across the end-Cretaceous boundary. Science 381, 1446–1451 (2023). 37769089 10.1126/science.adh3875

[24] P. R. Renne, A. L. Deino, F. J. Hilgen, K. F. Kuiper, D. F. Mark, W. S. Mitchell, L. E. Morgan, R. Mundil, J. Smit, Time scales of critical events around the Cretaceous-Paleogene boundary. Science 339, 684–687 (2013). 23393261 10.1126/science.1230492

[25] W. C. Clyde, J. Ramezani, K. R. Johnson, S. A. Bowring, M. J. Jones, Direct high-precision U-Pb geochronology of the end-Cretaceous extinction and calibration of Paleocene astronomical timescales. Earth Planet. Sci. Lett. 452, 272–280 (2016).

[26] B. Schoene, M. P. Eddy, K. M. Samperton, C. B. Keller, G. Keller, T. Adatte, S. F. R. Khadri, U-Pb constraints on pulsed eruption of the Deccan Traps across the end-Cretaceous mass extinction. Science 363, 862–866 (2019). 30792300 10.1126/science.aau2422

[27] B. Schoene, M. P. Eddy, C. B. Keller, K. M. Samperton, An evaluation of Deccan Traps eruption rates using geochronologic data. Geochronology 3, 181–198 (2021).

[28] J. W. H. Weijers, S. Schouten, J. C. van den Donker, E. C. Hopmans, J. S. Sinninghe Damsté, Environmental controls on bacterial tetraether membrane lipid distribution in soils. Geochim. Cosmochim. Acta 71, 703–713 (2007).

[29] C. De Jonge, E. C. Hopmans, C. I. Zell, J. H. Kim, S. Schouten, J. S. Sinninghe Damsté, Occurrence and abundance of 6-methyl branched glycerol dialkyl glycerol tetraethers in soils: Implications for palaeoclimate reconstruction. Geochim. Cosmochim. Acta 141, 97–112 (2014).

[30] B. D. A. Naafs, G. N. Inglis, Y. Zheng, M. J. Amesbury, H. Biester, R. Bindler, J. Blewett, M. A. Burrows, D. del Castillo Torres, F. M. Chambers, A. D. Cohen, Introducing global peat-specific temperature and pH calibrations based on brGDGT bacterial lipids. Geochim. Cosmochim. Acta 208, 285–301 (2017).

[31] L. K. O’Connor, E. D. Crampton-Flood, R. M. Jerrett, G. D. Price, B. D. A. Naafs, R. D. Pancost, P. McCormack, A. Lempotesis-Davies, B. E. van Dongen, S. K. Lengger, Steady decline in mean annual air temperatures in the first 30 ky after the Cretaceous-Paleogene boundary. Geology 51, 486–490 (2023).

[32] K. R. Johnson, D. J. Nichols, M. Attrep Jr., C. J. Orth, High-resolution leaf-fossil record spanning the Cretaceous/Tertiary boundary. Nature 340, 708–711 (1989).

[33] R. S. Barclay, K. R. Johnson, W. J. Betterton, D. L. Dilcher, Stratigraphy and megaflora of a K-T boundary section in the eastern Denver Basin, Colorado. Rocky Mt. Geol. 38, 45–71 (2003).

[34] C. J. Sprain, P. R. Renne, L. Vanderkluysen, K. Pande, S. Self, T. Mittal, The eruptive tempo of Deccan volcanism in relation to the Cretaceous-Paleogene boundary. Science 363, 866–870 (2019). 30792301 10.1126/science.aav1446

[35] K. J. Hsü, J. A. McKenzie, A “Strangelove” ocean in the earliest Tertiary. Geophys. Monogr. Ser. 32, 487–492 (1985).

[36] J. C. Zachos, M. P. Aubry, W. A. Berggren, T. Ehrendorfer, F. Heider, K. Lohmann, Chemobiostratigraphy of the Cretaceous/Paleocene boundary at Site 750, Southern Kerguelen Plateau. Proc. Sci. Results 120, 961–977 (1992).

[37] S. D’Hondt, P. Donaghay, J. C. Zachos, D. Luttenberg, M. Lindinger, Organic carbon fluxes and ecological recovery from the Cretaceous-Tertiary mass extinction. Science 282, 276–279 (1998). 9765149 10.1126/science.282.5387.276

[38] N. C. Arens, A. H. Jahren, Carbon isotope excursion in atmospheric CO_2_ at the Cretaceous-Tertiary boundary: Evidence from terrestrial sediments. Palaios 15, 314–322 (2000).

[39] D. Kroon, J. C. Zachos, C. Richter, P. Blum, J. Bowles, P. Gaillot, T. Hasegawa, E. C. Hathorne, D. A. Hodell, D. C. Kelly, J. H. Jung, Leg 208 synthesis: Cenozoic climate cycles and excursions. Proc. Ocean Drilling Prog. 10.2973/odp.proc.sr.208.201.2007, (2007).

[40] R. M. Jerrett, G. D. Price, S. T. Grimes, A. T. Dawson, A paleoclimatic and paleoatmospheric record from peatlands accumulating during the Cretaceous-Paleogene boundary event, Western Interior Basin, Canada. Geol. Soc. Am. Bull. 127, 1564–1582 (2015).

[41] J. S. Barnet, K. Littler, T. Westerhold, D. Kroon, M. J. Leng, I. Bailey, U. Röhl, J. C. Zachos, A high-fidelity benthic stable isotope record of Late Cretaceous–early Eocene climate change and carbon-cycling. Paleoceanogr. Paleoclimatol. 34, 672–691 (2019).

[42] F. Peterse, M. A. Prins, C. J. Beets, S. R. Troelstra, H. Zheng, Z. Gu, S. Schouten, J. S. Sinninghe Damsté, Decoupled warming and monsoon precipitation in East Asia over the last deglaciation. Earth Planet. Sci. Lett. 301, 256–264 (2011).

[43] T. S. Tobin, G. P. Wilson, J. M. Eiler, J. H. Hartman, Environmental change across a terrestrial Cretaceous-Paleogene boundary section in eastern Montana, USA, constrained by carbonate clumped isotope paleothermometry. Geology 42, 351–354 (2014).

[44] N. C. Arens, A. H. Jahren, R. Amundson, Can C3 plants faithfully record the isotopic composition of atmospheric carbon dioxide? Paleobiology 26, 137–164 (2000).

[45] J. S. Barnet, K. Littler, D. Kroon, M. J. Leng, T. Westerhold, U. Röhl, J. C. Zachos, A new high-resolution chronology for the late Maastrichtian warming event: Establishing robust temporal links with the onset of Deccan volcanism. Geology 46, 147–150 (2018).

[46] V. Gilabert, S. J. Batenburg, I. Arenillas, J. A. Arz, Contribution of orbital forcing and Deccan volcanism to global climatic and biotic changes across the Cretaceous-Paleogene boundary at Zumaia, Spain. Geology 50, 21–25 (2022).

[47] K. Caldeira, M. R. Rampino, Carbon dioxide emissions from Deccan volcanism and a K/T boundary greenhouse effect. Geophys. Res. Lett. 17, 1299–1302 (1990). 11538480 10.1029/gl017i009p01299

[48] T. S. Tobin, C. M. Bitz, D. Archer, Modeling climatic effects of carbon dioxide emissions from Deccan Traps volcanic eruptions around the Cretaceous–Paleogene boundary. Palaeogeogr. Palaeoclimatol. Palaeoecol. 478, 139–148 (2017).

[49] A. Hernandez Nava, B. A. Black, S. A. Gibson, R. J. Bodnar, P. R. Renne, L. Vanderkluysen, Reconciling early Deccan Traps CO_2_ outgassing and pre-KPB global climate. Proc. Natl. Acad. Sci. U.S.A. 118, e2007797118 (2021). 33782114 10.1073/pnas.2007797118 PMC8040825

[50] S. Self, The effects and consequences of very large explosive volcanic eruptions. Philos. Trans. A Math Phys. Eng. Sci. 364, 2073–2097 (2006). 16844649 10.1098/rsta.2006.1814

[51] H. Svensen, S. Planke, A. G. Polozov, N. Schmidbauer, F. Corfu, Y. Y. Podladchikov, B. Jamtveit, Siberian gas venting and the end Permian environmental crisis. Earth Planet. Sci. Lett. 277, 490–500 (2009).

[52] I. Aarnes, K. Fristad, S. Planke, H. Svensen, The impact of hostrock composition on devolatilization of sedimentary rocks during contact metamorphism around mafic sheet intrusions. Geochem. Geophys. Geosyst. 12, Q10019 (2011).

[53] M. P. Eddy, B. Schoene, K. M. Samperton, G. Keller, T. Adatte, S. F. Khadri, U-Pb zircon age constraints on the earliest eruptions of the Deccan Large Igneous Province, Malwa Plateau, India. Earth Planet. Sci. Lett. 540, 116249 (2020).

[54] S. V. Petersen, A. Dutton, K. C. Lohmann, End-Cretaceous extinction in Antarctica linked to both Deccan volcanism and meteorite impact via climate change. Nat. Commun. 7, 12079 (2016). 27377632 10.1038/ncomms12079 PMC4935969

[55] A. R. Sweet, D. R. Braman, J. F. Lerbekmo, Palynofloral response to K/T boundary events;A transitory interruption within a dynamic system, in *Global Catastrophes in Earth History; An Interdisciplinary Conference on Impacts, Volcanism, and Mass Mortality*, V. L. Sharpton,P. D. Ward, Eds. (Geological Society of America, 1990).

[56] M. T. Jones, D. A. Jerram, H. H. Svensen, C. Grove, The effects of large igneous provinces on the global carbon and sulfur cycles. Palaeogeogr. Palaeoclimatol. Palaeoecol. 441, 4–21 (2016).

[57] L. R. Kump, M. A. Arthur, Interpreting carbon-isotope excursions: Carbonates and organic matter. Chem. Geol. 161, 181–198 (1999).

[58] E. Mason, M. Edmonds, A. V. Turchyn, Remobilization of crustal carbon may dominate volcanic arc emissions. Science 357, 290–294 (2017). 28729507 10.1126/science.aan5049

[59] A. Robock, Volcanic eruptions and climate. Rev. Geophys. 38, 191–219 (2000).

[60] P. Delmelle, Environmental impacts of tropospheric volcanic gas plumes. Geol. Soc. Spec. Publ. 213, 381–399 (2003).

[61] I. M. Fendley, A. Fendley, T. Mittal, C. J. Sprain, M. Marvin-DiPasquale, T. S. Tobin, P. R. Renne, Constraints on the volume and rate of Deccan Traps flood basalt eruptions using a combination of high-resolution terrestrial mercury records and geochemical box models. Earth Planet. Sci. Lett. 524, 115721 (2019).

[62] A.-L. Chenet, F. Fluteau, V. Courtillot, M. Gérard, K. V. Subbarao, Determination of rapid Deccan eruptions across the Cretaceous-Tertiary boundary using paleomagnetic secular variation: Results from a 1200-m-thick section in the Mahabaleshwar escarpment. J. Geophys. Res. 113, B04101 (2008).

[63] D. J. Large, C. Marshall, Use of carbon accumulation rates to estimate the duration of coal seams and the influence of atmospheric dust deposition on coal composition. Geol. Soc. Spec. Publ. 404, 303–315 (2015).

[64] A. R. Sweet, B. D. Rickets, A. R. Cameron, D. K. Norris, An integrated analysis of the Brackett coal basin, Northwest Territories. Geol. Surv. Prof. Pap. 89, 85–99 (1989).

[65] D. J. Nichols, R. F. Fleming, Palynology and palynostratigraphy of Maastrichtian, Paleocene, and Eocene strata in the Denver Basin, Colorado. Rocky Mt. Geol. 37, 135–163 (2002).

[66] D. J. Nichols, K. R. Johnson, Palynology and microstratigraphy of Cretaceous-Tertiary boundary sections in southwestern North Dakota. Geol. Soc. Am. Spec. Paper 361, 95–143 (2002).

[67] E. C. Hopmans, J. W. Weijers, E. Schefuß, L. Herfort, J. S. Sinninghe Damsté, S. Schouten, A novel proxy for terrestrial organic matter in sediments based on branched and isoprenoid tetraether lipids. Earth Planet. Sci. Lett. 224, 107–116 (2004).

[68] C. De Jonge, E. E. Kuramae, D. Radujković, J. T. Weedon, I. A. Janssens, F. Peterse, The influence of soil chemistry on branched tetraether lipids in mid-and high latitude soils: Implications for brGDGT-based paleothermometry. Geochim. Cosmochim. Acta 310, 95–112 (2021).

[69] C. De Jonge, D. Radujković, B. D. Sigurdsson, J. T. Weedon, I. Janssens, F. Peterse, Lipid biomarker temperature proxy responds to abrupt shift in the bacterial community composition in geothermally heated soils. Org. Geochem. 137, 103897 (2019).

[70] D. J. van Hinsbergen, L. V. De Groot, S. J. van Schaik, W. Spakman, P. K. Bijl, A. Sluijs, C. G. Langereis, H. Brinkhuis, A paleolatitude calculator for paleoclimate studies. PLOS ONE 10, e0126946 (2015). 26061262 10.1371/journal.pone.0126946 PMC4462584

[71] C. Diessel, R. Boyd, J. Wadsworth, D. Leckie, G. Chalmers, On balanced and unbalanced accommodation/peat accumulation ratios in the Cretaceous coals from Gates Formation, Western Canada, and their sequence-stratigraphic significance. Int. J. Coal Geol. 43, 143–186 (2003).

[72] E. C. Hopmans, S. Schouten, J. S. Sinninghe Damsté, The effect of improved chromatography on GDGT-based palaeoproxies. Org. Geochem. 93, 1–6 (2016).

[73] S. Schouten, E. C. Hopmans, J. S. Sinninghe Damsté, The effect of maturity and depositional redox conditions on archaeal tetraether lipid palaeothermometry. Org. Geochem. 35, 567–571 (2004).

[74] J. W. H. Weijers, P. Steinmann, E. C. Hopmans, S. Schouten, J. S. Sinninghe Damsté, Bacterial tetraether membrane lipids in peat and coal: Testing the MBT–CBT temperature proxy for climate reconstruction. Org. Geochem. 42, 477–486 (2011).

[75] A. Sluijs, J. Frieling, G. N. Inglis, K. G. Nierop, F. Peterse, F. Sangiorgi, S. Schouten, Late Paleocene–early Eocene Arctic Ocean sea surface temperatures: Reassessing biomarker paleothermometry at Lomonosov Ridge. Clim. Past 16, 2381–2400 (2020).

[76] C. De Jonge, A. Stadnitskaia, E. C. Hopmans, G. Cherkashov, A. Fedotov, J. S. Sinninghe Damsté, In situ produced branched glycerol dialkyl glycerol tetraethers in suspended particulate matter from the Yenisei River, Eastern Siberia. Geochim. Cosmochim. Acta 125, 476–491 (2014).

[77] P. D. Hughes, G. Mallon, A. Brown, H. J. Essex, J. D. Stanford, S. Hotes, The impact of high tephra loading on late-Holocene carbon accumulation and vegetation succession in peatland communities. Quat. Sci. Rev. 67, 160–175 (2013).

[78] J. L. Ratcliffe, D. J. Lowe, L. A. Schipper, M. J. Gehrels, A. D. French, D. I. Campbell, Rapid carbon accumulation in a peatland following Late Holocene tephra deposition. Quat. Sci. Rev. 246, 106505 (2020).

[79] R. Payne, J. Blackford, Distal volcanic impacts on peatlands: Palaeoecological evidence from Alaska. Quat. Sci. Rev. 27, 2012–2030 (2008).

[80] S. S. Crowley, D. A. Dufek, R. W. Stanton, T. A. Ryer, The effects of volcanic ash disturbances on a peat: Forming environment: Environmental disruption and taphonomic consequences. Palaios 9, 158–174 (1994).

[81] S. S. Dirghangi, M. Pagani, M. T. Hren, B. J. Tipple, Distribution of glycerol dialkyl glycerol tetraethers in soils from two environmental transects in the USA. Org. Geochem. 59, 49–60 (2013).

[82] C. I. Blaga, G. J. Reichart, O. Heiri, J. S. Sinninghe Damsté, Tetraether membrane lipid distributions in water-column particulate matter and sediments: A study of 47 European lakes along a north–south transect. J. Paleolimnol. 41, 523–540 (2009).

[83] E. D. Crampton-Flood, J. E. Tierney, F. Peterse, F. M. Kirkels, J. S. Sinninghe Damsté, Global soil and peat branched GDGT compilation dataset. PANGAEA (2019); 10.1594/PANGAEA.907818.

