# Supplementary material for: Terrestrial evidence for volcanogenic sulfate-driven cooling event ~30 kyr before the Cretaceous–Paleogene mass extinction
Source: Sci Adv. 2024 Dec 18;10(51):eado5478. doi: 10.1126/sciadv.ado5478 (PMC11654674; doi:10.1126/sciadv.ado5478)
Supplement: Supplementary file 1 — Supplementary Text Figs. S1 and S2 Legend for data S1 References [file sciadv.ado5478_sm.pdf]

Science Advances
AAAS

Supplementary Materials for

Terrestrial evidence for volcanogenic sulfate-driven cooling event ~30 kyr before the Cretaceous–Paleogene mass extinction

Lauren K. O'Connor *et al.*

Corresponding author: Lauren K. O'Connor, l.k.oconnor@uu.nl

Sci. Adv. **10**, eado5478 (2024)
DOI: 10.1126/sciadv.ado5478

The PDF file includes:

Supplementary Text
Figs. S1 and S2
Legend for data S1
References

Other Supplementary Material for this manuscript includes the following:

Data S1

Supplementary Text

Site description and sampling

At the time of the K–Pg boundary, West Bijou (Colorado, 39°34′14″N, 104°18′09″W) and Pyramid Butte (North Dakota, 46°25′03″N, 103°58′33″W) were located at approximately 45°N and 51°N, respectively (70), in the foreland basins of the Rocky Mountains (40), at negligible altitude. At West Bijou, the K–Pg boundary occurs 45 cm above the base of 122 cm thick lignite in the Denver Formation, Denver Basin. It is defined by a palynological extinction and coincides with the top of a 3–4cm-thick mudstone containing an iridium anomaly, and shocked quartz (33). The lignite interstratifies with numerous 1.5 cm to 15 cm-thick rhyolitic tuffs, particularly above the K–Pg boundary where they are, in places, bioturbated through the lignite. At Pyramid Butte, the K–Pg boundary occurs 90.5 cm above the base of a lignite, the latter of which defines the base of the Fort Union Formation, Williston Basin. The boundary is defined by a palynological extinction (66) and coincides with the top of a 0.5 cm thick mudstone containing an iridium anomaly and shocked minerals (32). The mudstone caps the lignite and is overlain by a discontinuous (0–10 cm-thick), bioturbated sandstone, wherein burrows extend up to 5 cm downwards through the iridium-enriched mudstone and upper part of the lignite. Note that Johnson et al. (32) and Nichols & Johnson (66) place the K–Pg boundary at 95.0 cm above the base of the lignite, the difference representing lateral variation in the thickness of the underlying lignite.

At both sites, the lignite surface was excavated by 1 m to limit the effects of weathering on subsequent analyses. Prior to sampling, a lignite lithotype log was produced using the classification scheme of Diessel (71). Contiguous lignite blocks, representing the whole coal seam stratigraphy were collected (Data S1). Whenever possible, the specimens were removed intact, and their younging direction was recorded, such that their internal stratigraphy could be preserved. The samples were wrapped in aluminium foil for transport to the University of Manchester (UK), and then freeze-dried and powdered prior to geochemical analysis.

Lipid biomarker analysis

Approximately 0.5 g of powdered sediment was solvent extracted using 20mL of DCM/methanol (9:1, v/v) in a microwave assisted reactor system (MARS 6, CEM) at the University of Manchester. The temperature in the microwave was programmed to increase linearly from room temperature to 70°C over 10 min at which it was held for 10 min, and then cooled to 25°C over 20 minutes. The total lipid extract was separated into apolar and polar fractions by column chromatography, using hexane/DCM (9:1, v/v) and DCM/methanol (1:1, v/v), respectively, as the eluents and activated Al_2O_3 as the stationary phase. The polar fraction was re-dissolved in hexane/propanol (99:1, v/v) and filtered using a 0.45 μm PTFE filter prior to analysis.

Samples were analyzed for their GDGT content using a Dionex LPG-U3400(SDN) UHPLC liquid chromatography system and Thermo Scientific Q Exactive Focus mass spectrometer with Atmospheric Pressure Chemical Ionisation (APCI) at the University of Plymouth (31). For the detection of GDGTs, a modified method of Hopmans et al. (72) was used as described in detail in O'Connor et al. (31). The MBT'_{5Me} index was calculated following De Jonge et al. (29):

$$MBT'_{5me} = (Ia + Ib + Ic)/(Ia + Ib + Ic + IIa + IIb + IIc + IIIa)$$

The average standard deviation for the MBT'_{5Me} index values based on repeat measurements of an in-house GDGT standard was 0.026 units. The data are reported in Supplementary Data S1.

The peat specific calibration ($MAAT_{peat}$, 30) was used to convert MBT'_{5me} values into mean annual air temperatures:

$$MAAT_{peat}\ (°C) = 52.18(MBT'_{5Me}) - 23.05$$

To scrutinize the samples for non-thermal influences on GDGT production/preservation (as discussed below) the branched and isoprenoid tetraether (BIT; 68) index, the isomer ratio (IR ratio; relative abundance of 6-methyl versus 5-methyl brGDGTs; 69) and the degree of cyclization (DC; 69) and community index (CI; 70) were calculated:

$$BIT\ index = (Ia + IIa + IIa' + IIIa + IIIa')/(Ia + IIa + IIa' + IIIa + IIIa' + cren)$$

$$DC = (Ib + IIb)/(Ia + Ib + IIa + IIb)$$

$$IR = (IIa' + IIIa')/(IIa + IIa' + IIIa + IIIa')$$

$$CI = Ia/(Ia + IIa + IIIa)$$

Sample filtering and scrutiny

Thermal maturity, lithological and ecological change may exert non-thermal influences on GDGT production and preservation, biasing temperature reconstructions (73, 74). As such, the brGDGT distributions in all samples should be screened to ensure the fidelity of temperature signal that they reflect. Considering that all samples are immature lignites, with no obvious ecological issues and that there is no clear-cut framework for filtering anomalous brGDGT distributions, such as exists for the TEX_{86} proxy for sea-surface temperature reconstruction based on isoprenoid GDGTs produced by marine archaea (see assessment of confounding factors in Sluijs et al. (75)), we first evaluate the stratigraphic changes in brGDGT distributions using the IR and DC that capture the relative abundance of 6-methylated brGDGT isomers and the degree of cyclization of 5-methyl brGDGTs, respectively, in relation to the MBT'5me. Both membrane adjustments are positively related to changes in pH in modern soils and peats (76): however, in the section above the K–Pg boundary, IR remains stable whereas the DC starts to increase (Fig. S1), suggesting the influence of an additional environmental driver. Interestingly, this interval is characterized by the presence of tuffs. From modern peat environments, volcanic tuff deposits are known to disrupt carbon cycling (77, 78) and the microbial community (79) and may also introduce an impermeable layer within the stratigraphy that can affect the peat hydrology (80). Such disturbances of the peat microbial community and hydrology are reflected by the large change in the CI and BIT index, respectively, above the boundary and into the tuff-affected layers (Fig. S1). A decrease in BIT is the effect of an increase in the proportion of the isoGDGT crenarchaeol relative to brGDGTs and has been linked to both arid conditions, facilitating the growth of archaea over bacteria in mineral soils (81), and wetter conditions where crenarchaeol is preferentially produced in more lacustrine like settings (82). Although both scenarios are seemingly not immediately projectable on the lignites studied here, the BIT index shows a similar trend close to the tuff at -43 cm in West Bijou, suggesting that the deposition of tuffs equally perturbated the microbial community at that time.

Following the inference that the microbial community was affected by tuffs, and to further assess the reliability of the brGDGT-temperature relationship in the lignites, we compared the relative abundance of major brGDGTs Ia and IIa in the lignite sections with those in the modern environment (obtained from Crampton-Flood et al. (83)), where soils are grouped into global clusters broadly based on temperature and pH (68, 69). All Pyramid Butte samples plot within the range of warm and acidic modern soils (Fig. S2), in accordance with the expected environmental conditions of Cretaceous climate and the pH of peat deposits. By contrast, only

some of the West Bijou samples plot within this same group, while others plot more with cool but more importantly alkaline soils that in the modern environment have a distinct microbial community and possibly responds to temperature differently (68). Notably, those samples are all taken from within ~5–10 cm of a tuff (Figs. S1 and S2) and correspond with the anomalous trends in brGDGT indices as well as BIT index values <0.9, indicating that this anomalous behavior can be linked to the presence of tuff rather than actual changes in temperature.

Therefore, we excluded all samples within 5 cm of a tuff and/or a BIT index value <0.95 from our temperature reconstruction (Supplementary Data S1). The approach of examining community distributions as a standard part of data assessment, and thus the reliability of the final temperature record, is recommended. However, we suggest caution in applying the same cut-offs to other sections, as we cannot determine yet how much of this effect is localized to the sites analyzed in this study.

$\delta^{13}C$ analysis

Bulk-organic carbon-isotope analyses were conducted at the University of Plymouth using a Thermo Scientific Delta V Advantage. Carbon-isotope ratios are expressed using the internationally accepted per mil (‰) standard notation relative to the Vienna Peedee belemnite (VPDB) standard (Supplementary Data S1). Instrument calibration was achieved using three international standards: USGS 0 (l-glutamic acid, $\delta^{13}C$ = -26.389‰), USGS24 (graphite, $\delta^{13}C$ = -16.049‰), and IAEACH-7 (polyethylene, $\delta^{13}C$ = -32.151‰). Standard deviations for replicate analyses of international standard USGS54, in-house standard IWC01, and procedural duplicate PB026.5 were 0.04, 0.08, and 0.05 respectively.

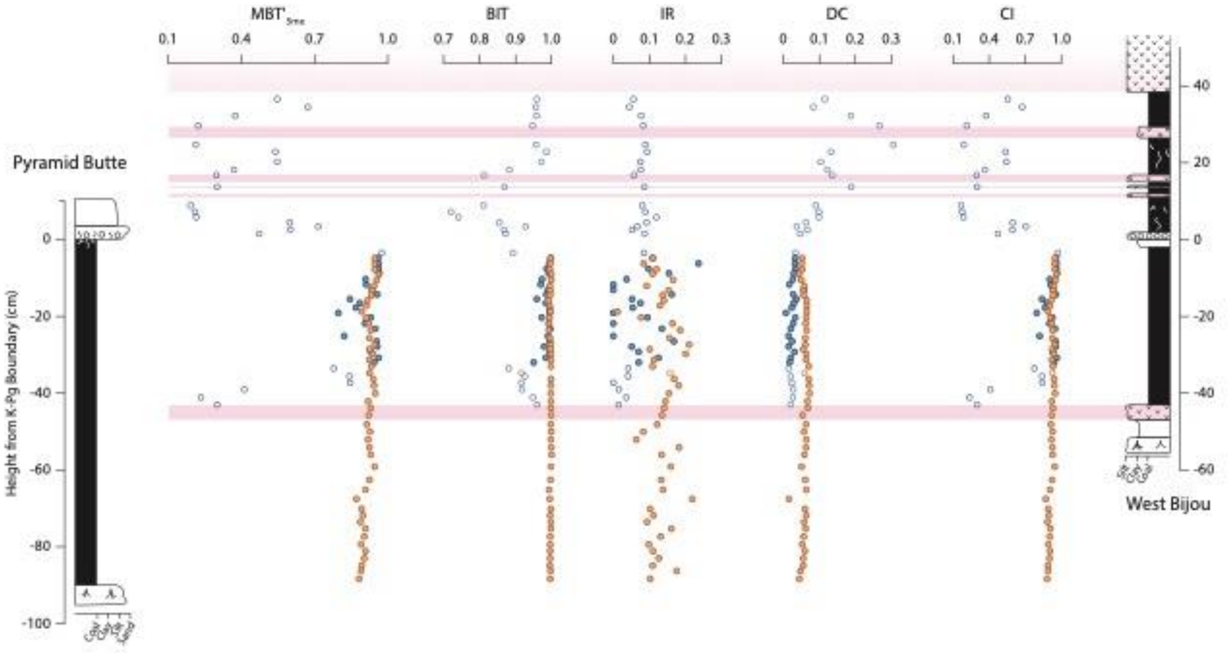

Fig. S1.

GDGT indices from Pyramid Butte (orange) and West Bijou (blue) plotted against depth in cm relative to the K–Pg boundary. Lithological logs of both sites are also shown, with tuffs at West Bijou highlighted in pink. Closed circles show samples used to reconstruct paleotemperatures from MBT'_{5Me} values, after the exclusion of samples biased by non-thermal influences. Potential non-thermal influences on GDGTs appear related to tuff layers, as evident from anomalous values in several GDGT indices: branched and isoprenoid tetraether (BIT) index, relative abundance of 6-methyl versus 5-methyl brGDGTs (IR), degree of cyclization (DC), and community index (CI).

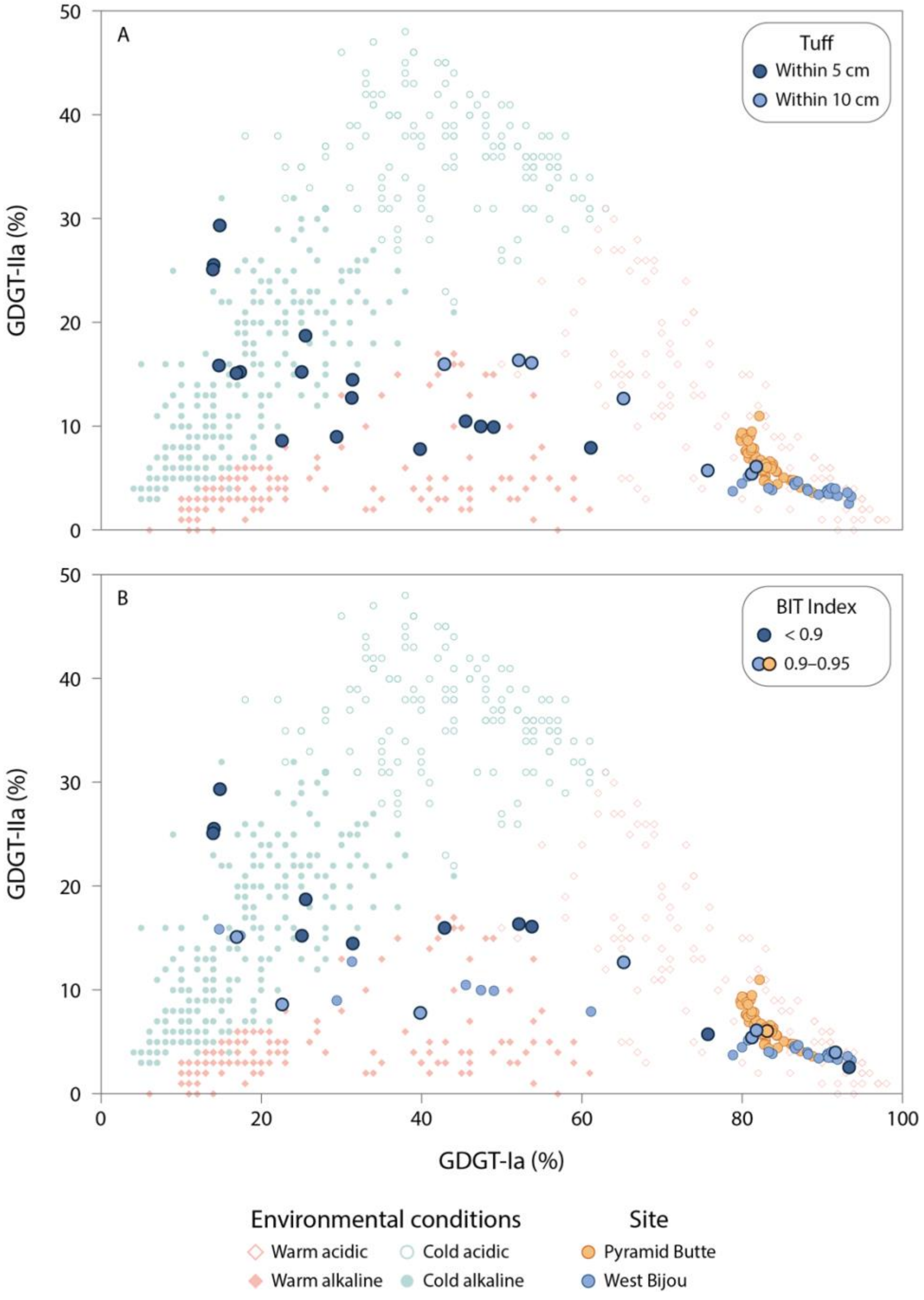

Fig. S2.

Fractional abundances of Ia and IIa brGDGTs. Fractional abundances of Ia and IIa brGDGTs from a compilation of modern samples (obtained from Crampton-Flood et al. (83)) overlaid by samples from Pyramid Butte and West Bijou (larger circles). Panel A highlights samples within 0–5 cm (dark circles) or 5–10 cm (dark outlined circles) of a tuff (see Fig. S1 for stratigraphic context). Panel (B) highlights samples with BIT values <0.9 (dark circles) or 0.9–0.95 (dark outlined circles).

Data S1. (separate file)

Stratigraphic and lithological data, glycerol dialkyl glycerol tetraethers (GDGT) absolute and relative abundances, palaeothermometry (air temperature reconstructions) and other calibrated environmental parameter calculations, and bulk-organic carbon-isotope ($\delta^{13}C$) data from samples from West Bijou and Pyramid Butte. Samples are listed stratigraphically for each site, from the top of the section to the bottom. The site name is given (column A), the University of Manchester laboratory identifier of the (sub)sample (column B), the stratigraphic height relative to the K–Pg boundary in cm (columns C–E), age relative to the K–Pg boundary in ka (column F), the sample bulk lithology (column G), and the stratigraphic distance in cm of the sample relative to a volcanic tuff (column H). $\delta^{13}C$ values and duplicate/mean (where appropriate) are reported in permil (‰) VPDB (columns I–K). Chromatogram peak areas of isoprenoidal GDGTs are given in columns L to Q, and their sum in column AB. Chromatogram peak areas of branched GDGTs are given in columns R to AB, and their sum in column AD. The fractional abundances of these GDGTs are also reported (columns AE–AO). The following calculated indices, are also provided: MBT'_{5Me} (column AP) calculated from De Jonge et al. (29); mean annual air temperature (MAAT; column AQ) calculated from Naafs et al. (30); branched:isoprenoid tetraether (BIT; column AR) calculated from Hopmans et al. (67); degree of cyclization (DC; column AS) calculated from De Jonge et al. (68); relative abundance of 6-methyl versus 5-methyl brGDGTs (IR; column AT) calculated from De Jonge et al. (68); and community index (CI; column AU) calculated from De Jonge et al. (69). Blank cells (columns L–AD) denote samples not analysed for GDGTs or those with concentrations below the detection limit/unquantifiable GDGTs.

REFERENCES AND NOTES

1. N. MacLeod, P. F. Rawson, P. L. Forey, F. T. Banner, M. K. Boudagher-Fadel, P. R. Bown, J. A. Burnett, P. Chambers, S. Culver, S. E. Evans, C. Jeffery, M. A. Kaminski, A. R. Lord, A. C. Milner, A. R. Milner, N. Morris, E. Owen, B. R. Rosen, A. B. Smith, P. D. Taylor, E. Urquhart, J. R. Young, The Cretaceous-Tertiary biotic transition. *J. Geol. Soc.* **154**, 265–292 (1997).

2. M. A. O'Leary, J. I. Bloch, J. J. Flynn, T. J. Gaudin, A. Giallombardo, N. P. Giannini, S. L. Goldberg, B. P. Kraatz, Z.-X. Luo, J. Meng, X. Ni, M. J. Novacek, F. A. Perini, Z. S. Randall, G. W. Rougier, E. J. Sargis, M. T. Silcox, N. B. Simmons, M. Spaulding, P. M. Velazco, M. Weksler, J. R. Wible, A. L. Cirranello, The placental mammal ancestor and the post–K–Pg radiation of placentals. *Science* **339**, 662–667 (2013).

3. P. Hull, Life in the aftermath of mass extinctions. *Curr. Biol.* **25**, R941–R952 (2015).

4. L. W. Alvarez, W. Alvarez, F. Asro, H. V. Michel, Extraterrestrial cause for the Cretaceous-Tertiary extinction. *Science* **208**, 1095–1108 (1980).

5. A. R. Hildebrand, G. T. Penfield, D. A. Kring, M. Pilkington, A. Camargo, S. B. Jacobsen, W. V. Boynton, Chicxulub crater—A possible Cretaceous-Tertiary boundary impact crater on the Yucatan Peninsula, Mexico. *Geology* **19**, 867–871 (1991).

6. P. Schulte, L. Alegret, I. Arenillas, J. A. Arz, P. J. Barton, P. R. Bown, T. J. Bralower, G. L. Christeson, P. Claeys, C. S. Cockell, G. S. Collins, The Chicxulub asteroid impact and mass extinction at the Cretaceous-Paleogene boundary. *Science* **327**, 1214–1218 (2010).

7. V. Courtillot, J. Besse, D. Vandamme, R. Montigny, J. J. Jaeger, H. Cappetta, Deccan flood basalt at the Cretaceous/Tertiary boundary? *Earth Planet. Sci. Lett.* **80**, 361–374 (1986).

8. R. A. Duncan, D. G. Pyle, Rapid eruption of the Deccan flood basalts at the Cretaceous/Tertiary boundary. *Nature* **333**, 841–843 (1988).

9. V. Courtillot, F. Fluteau, Cretaceous extinctions: The volcanic hypothesis. *Science* **328**, 973–974 (2010).

10. B. Gertsch, G. Keller, T. Adatte, R. Garg, V. Prasad, Z. Berner, D. Fleitmann, Environmental effects of Deccan volcanism across the Cretaceous-Tertiary transition in Meghalaya, India. *Earth Planet. Sci. Lett.* **310**, 272–285 (2011).

11. E. Font, T. Adatte, A. N. Sial, L. D. de Lacerda, G. Keller, J. Punekar, Mercury anomaly, Deccan volcanism, and the end-Cretaceous mass extinction. *Geology* **44**, 171–174 (2016).

12. G. Keller, P. Mateo, J. Monkenbusch, N. Thibault, J. Punekar, J. E. Spangenberg, S. Abramovich, S. Ashckenazi-Polivoda, B. Schoene, M. P. Eddy, K. M. Samperton, Mercury linked to Deccan Traps volcanism, climate change and the end-Cretaceous mass extinction. *Global Planet. Change* **194**, 103312 (2020).

13. K. O. Pope, K. H. Baines, A. C. Ocampo, B. A. Ivanov, Impact winter and the Cretaceous/Tertiary extinctions: Results of a Chicxulub asteroid impact model. *Earth Planet. Sci. Lett.* **128**, 719–725 (1994).

14. C. G. Bardeen, R. R. Garcia, O. B. Toon, A. J. Conley, On transient climate change at the Cretaceous-Paleogene boundary due to atmospheric soot injections. *Proc. Natl. Acad. Sci. U.S.A.* **114**, E7415–E7424 (2017).

15. J. Brugger, G. Feulner, S. Petri, Baby, it's cold outside: Climate model simulations of the effects of the asteroid impact at the end of the Cretaceous. *Geophys. Res. Lett.* **44**, 419–427 (2017).

16. C. B. Senel, P. Kaskes, O. Temel, J. Vellekoop, S. Goderis, R. DePalma, M. A. Prins, P. Claeys, Ö. Karatekin, Chicxulub impact winter sustained by fine silicate dust. *Nat. Geosci.* **16**, 1033–1040 (2013).

17. C. B. Officer, A. Hallam, C. L. Drake, J. D. Devine, Late Cretaceous and paroxysmal Cretaceous/Tertiary extinctions. *Nature* **326**, 143–149 (1987).

18. J. D. O'Keefe, T. J. Ahrens, Impact production of CO_2 by the Cretaceous/Tertiary extinction bolide and the resultant heating of the Earth. *Nature* **338**, 247–249 (1989).

19. S. Self, A. Schmidt, T. A. Mather, Emplacement characteristics, time scales, and volcanic gas release rates of continental flood basalt eruptions on Earth. *Geol. Soc. Am. Spec. Pap.* **505**, 319–337 (2014).

20. C. Dessert, B. Dupre, L. M. Francois, J. Schott, J. Gaillardet, G. Chakrapani, S. Bajpai, Erosion of Deccan Traps determined by river geochemistry: Impact on the global climate and the Sr-87/Sr-86 ratio of seawater. *Earth Planet. Sci. Lett.* **188**, 459–474 (2001).

21. A. Schmidt, R. A. Skeffington, T. Thordarson, S. Self, P. M. Forster, A. Rap, A. Ridgwell, D. Fowler, M. Wilson, G. W. Mann, P. B. Wignall, Selective environmental stress from sulfur emitted by continental flood basalt eruptions. *Nat. Geosci.* **9**, 77–82 (2016).

22. S. Callegaro, D. R. Baker, P. R. Renne, L. Melluso, K. Geraki, M. J. Whitehouse, A. De Min, A. Marzoli, Recurring volcanic winters during the latest Cretaceous: Sulfur and fluorine budgets of Deccan Traps lavas. *Sci. Adv.* **9**, eadg8284 (2023).

23. A. A. Cox, C. B. Keller, A Bayesian inversion for emissions and export productivity across the end-Cretaceous boundary. *Science* **381**, 1446–1451 (2023).

24. P. R. Renne, A. L. Deino, F. J. Hilgen, K. F. Kuiper, D. F. Mark, W. S. Mitchell, L. E. Morgan, R. Mundil, J. Smit, Time scales of critical events around the Cretaceous-Paleogene boundary. *Science* **339**, 684–687 (2013).

25. W. C. Clyde, J. Ramezani, K. R. Johnson, S. A. Bowring, M. J. Jones, Direct high-precision U-Pb geochronology of the end-Cretaceous extinction and calibration of Paleocene astronomical timescales. *Earth Planet. Sci. Lett.* **452**, 272–280 (2016).

26. B. Schoene, M. P. Eddy, K. M. Samperton, C. B. Keller, G. Keller, T. Adatte, S. F. R. Khadri, U-Pb constraints on pulsed eruption of the Deccan Traps across the end-Cretaceous mass extinction. *Science* **363**, 862–866 (2019).

27. B. Schoene, M. P. Eddy, C. B. Keller, K. M. Samperton, An evaluation of Deccan Traps eruption rates using geochronologic data. *Geochronology* **3**, 181–198 (2021).

28. J. . W. Weijers, S. Schouten, J. C. van den Donker, E. C. Hopmans, J. S. Sinninghe Damsté, Environmental controls on bacterial tetraether membrane lipid distribution in soils. *Geochim. Cosmochim. Acta* **71**, 703–713 (2007).

29. C. De Jonge, E. C. Hopmans, C. I. Zell, J. H. Kim, S. Schouten, J. S. Sinninghe Damsté, Occurrence and abundance of 6-methyl branched glycerol dialkyl glycerol tetraethers in soils: Implications for palaeoclimate reconstruction. *Geochim. Cosmochim. Acta* **141**, 97–112 (2014).

30. B. D. A. Naafs, G. N. Inglis, Y. Zheng, M. J. Amesbury, H. Biester, R. Bindler, J. Blewett, M. A. Burrows, D. del Castillo Torres, F. M. Chambers, A. D. Cohen, Introducing global peat-specific temperature and pH calibrations based on brGDGT bacterial lipids. *Geochim. Cosmochim. Acta* **208**, 285–301 (2017).

31. L. K. O'Connor, E. D. Crampton-Flood, R. M. Jerrett, G. D. Price, B. D. A. Naafs, R. D. Pancost, P. McCormack, A. Lempotesis-Davies, B. E. van Dongen, S. K. Lengger, Steady decline in mean annual air temperatures in the first 30 ky after the Cretaceous-Paleogene boundary. *Geology* **51**, 486–490 (2023).

32. K. R. Johnson, D. J. Nichols, M. Attrep Jr., C. J. Orth, High-resolution leaf-fossil record spanning the Cretaceous/Tertiary boundary. *Nature* **340**, 708–711 (1989).

33. R. S. Barclay, K. R. Johnson, W. J. Betterton, D. L. Dilcher, Stratigraphy and megaflora of a K-T boundary section in the eastern Denver Basin, Colorado. *Rocky Mt. Geol.* **38**, 45–71 (2003).

34. C. J. Sprain, P. R. Renne, L. Vanderkluysen, K. Pande, S. Self, T. Mittal, The eruptive tempo of Deccan volcanism in relation to the Cretaceous-Paleogene boundary. *Science* **363**, 866–870 (2019).

35. K. J. Hsü, J. A. McKenzie, A "Strangelove" ocean in the earliest Tertiary. *Geophys. Monogr. Ser.* **32**, 487–492 (1985).

36. J. C. Zachos, M. P. Aubry, W. A. Berggren, T. Ehrendorfer, F. Heider, K. Lohmann, Chemobiostratigraphy of the Cretaceous/Paleocene boundary at Site 750, Southern Kerguelen Plateau. *Proc. Sci. Results* **120**, 961–977 (1992).

37. S. D'Hondt, P. Donaghay, J. C. Zachos, D. Luttenberg, M. Lindinger, Organic carbon fluxes and ecological recovery from the Cretaceous-Tertiary mass extinction. *Science* **282**, 276–279 (1998).

38. N. C. Arens, A. H. Jahren, Carbon isotope excursion in atmospheric CO_2 at the Cretaceous-Tertiary boundary: Evidence from terrestrial sediments. *Palaios* **15**, 314–322 (2000).

39. D. Kroon, J. C. Zachos, C. Richter, P. Blum, J. Bowles, P. Gaillot, T. Hasegawa, E. C. Hathorne, D. A. Hodell, D. C. Kelly, J. H. Jung, Leg 208 synthesis: Cenozoic climate cycles and excursions. *Proc. Ocean Drilling Prog.* 10.2973/odp.proc.sr.208.201.2007 , (2007).

40. R. M. Jerrett, G. D. Price, S. T. Grimes, A. T. Dawson, A paleoclimatic and paleoatmospheric record from peatlands accumulating during the Cretaceous-Paleogene boundary event, Western Interior Basin, Canada. *Geol. Soc. Am. Bull.* **127**, 1564–1582 (2015).

41. J. S. Barnet, K. Littler, T. Westerhold, D. Kroon, M. J. Leng, I. Bailey, U. Röhl, J. C. Zachos,A high-fidelity benthic stable isotope record of Late Cretaceous–early Eocene climate change and carbon-cycling. *Paleoceanogr. Paleoclimatol.* **34**, 672–691 (2019).

42. F. Peterse, M. A. Prins, C. J. Beets, S. R. Troelstra, H. Zheng, Z. Gu, S. Schouten, J. S. Sinninghe Damsté, Decoupled warming and monsoon precipitation in East Asia over the last deglaciation. *Earth Planet. Sci. Lett.* **301**, 256–264 (2011).

43. T. S. Tobin, G. P. Wilson, J. M. Eiler, J. H. Hartman, Environmental change across a terrestrial Cretaceous-Paleogene boundary section in eastern Montana, USA, constrained by carbonate clumped isotope paleothermometry. *Geology* **42**, 351–354 (2014).

44. N. C. Arens, A. H. Jahren, R. Amundson, Can C3 plants faithfully record the isotopic composition of atmospheric carbon dioxide? *Paleobiology* **26**, 137–164 (2000).

45. J. S. Barnet, K. Littler, D. Kroon, M. J. Leng, T. Westerhold, U. Röhl, J. C. Zachos, A new high-resolution chronology for the late Maastrichtian warming event: Establishing robust temporal links with the onset of Deccan volcanism. *Geology* **46**, 147–150 (2018).

46. V. Gilabert, S. J. Batenburg, I. Arenillas, J. A. Arz, Contribution of orbital forcing and Deccan volcanism to global climatic and biotic changes across the Cretaceous-Paleogene boundary at Zumaia, Spain. *Geology* **50**, 21–25 (2022).

47. K. Caldeira, M. R. Rampino, Carbon dioxide emissions from Deccan volcanism and a K/T boundary greenhouse effect. *Geophys. Res. Lett.* **17**, 1299–1302 (1990).

48. T. S. Tobin, C. M. Bitz, D. Archer, Modeling climatic effects of carbon dioxide emissions from Deccan Traps volcanic eruptions around the Cretaceous–Paleogene boundary. *Palaeogeogr. Palaeoclimatol. Palaeoecol.* **478**, 139–148 (2017).

49. A. Hernandez Nava, B. A. Black, S. A. Gibson, R. J. Bodnar, P. R. Renne, L. Vanderkluysen, Reconciling early Deccan Traps CO_2 outgassing and pre-KPB global climate. *Proc. Natl. Acad. Sci. U.S.A.* **118**, e2007797118 (2021).

50. S. Self, The effects and consequences of very large explosive volcanic eruptions. *Philos. Trans. A Math Phys. Eng. Sci.* **364**, 2073–2097 (2006).

51. H. Svensen, S. Planke, A. G. Polozov, N. Schmidbauer, F. Corfu, Y. Y. Podladchikov, B. Jamtveit, Siberian gas venting and the end Permian environmental crisis. *Earth Planet. Sci. Lett.* **277**, 490–500 (2009).

52. I. Aarnes, K. Fristad, S. Planke, H. Svensen, The impact of hostrock composition on devolatilization of sedimentary rocks during contact metamorphism around mafic sheet intrusions. *Geochem. Geophys. Geosyst.* **12**, Q10019 (2011).

53. M. P. Eddy, B. Schoene, K. M. Samperton, G. Keller, T. Adatte, S. F. Khadri, U-Pb zircon age constraints on the earliest eruptions of the Deccan Large Igneous Province, Malwa Plateau, India. *Earth Planet. Sci. Lett.* **540**, 116249 (2020).

54. S. V. Petersen, A. Dutton, K. C. Lohmann, End-Cretaceous extinction in Antarctica linked to both Deccan volcanism and meteorite impact via climate change. *Nat. Commun.* **7**, 12079 (2016).

55. A. R. Sweet, D. R. Braman, J. F. Lerbekmo, Palynofloral response to K/T boundary events;A transitory interruption within a dynamic system, in *Global Catastrophes in Earth History; An Interdisciplinary Conference on Impacts, Volcanism, and Mass Mortality*, V. L. Sharpton,P. D. Ward, Eds. (Geological Society of America, 1990).

56. M. T. Jones, D. A. Jerram, H. H. Svensen, C. Grove, The effects of large igneous provinces on the global carbon and sulfur cycles. *Palaeogeogr. Palaeoclimatol. Palaeoecol.* **441**, 4–21 (2016).

57. L. R. Kump, M. A. Arthur, Interpreting carbon-isotope excursions: Carbonates and organic matter. *Chem. Geol.* **161**, 181–198 (1999).

58. E. Mason, M. Edmonds, A. V. Turchyn, Remobilization of crustal carbon may dominate volcanic arc emissions. *Science* **357**, 290–294 (2017).

59. A. Robock, Volcanic eruptions and climate. *Rev. Geophys.* **38**, 191–219 (2000).

60. P. Delmelle, Environmental impacts of tropospheric volcanic gas plumes. *Geol. Soc. Spec. Publ.* **213**, 381–399 (2003).

61. I. M. Fendley, A. Fendley, T. Mittal, C. J. Sprain, M. Marvin-DiPasquale, T. S. Tobin, P. R. Renne, Constraints on the volume and rate of Deccan Traps flood basalt eruptions using a combination of high-resolution terrestrial mercury records and geochemical box models. *Earth Planet. Sci. Lett.* **524**, 115721 (2019).

62. A.-L. Chenet, F. Fluteau, V. Courtillot, M. Gérard, K. V. Subbarao, Determination of rapid Deccan eruptions across the Cretaceous-Tertiary boundary using paleomagnetic secular variation: Results from a 1200-m-thick section in the Mahabaleshwar escarpment.*J. Geophys. Res.* **113**, B04101 (2008).

63. D. J. Large, C. Marshall, Use of carbon accumulation rates to estimate the duration of coal seams and the influence of atmospheric dust deposition on coal composition. *Geol. Soc. Spec. Publ.* **404**, 303–315 (2015).

64. A. R. Sweet, B. D. Rickets, A. R. Cameron, D. K. Norris, An integrated analysis of the Brackett coal basin, Northwest Territories. *Geol. Surv. Prof. Pap.* **89**, 85–99 (1989).

65. D. J. Nichols, R. F. Fleming, Palynology and palynostratigraphy of Maastrichtian, Paleocene, and Eocene strata in the Denver Basin, Colorado. *Rocky Mt. Geol.* **37**, 135–163 (2002).

66. D. J. Nichols, K. R. Johnson, Palynology and microstratigraphy of Cretaceous-Tertiary boundary sections in southwestern North Dakota. *Geol. Soc. Am. Spec. Paper* **361**, 95–143 (2002).

67. E. C. Hopmans, J. W. Weijers, E. Schefuß, L. Herfort, J. S. Sinninghe Damsté, S. Schouten,A novel proxy for terrestrial organic matter in sediments based on branched and isoprenoid tetraether lipids. *Earth Planet. Sci. Lett.* **224**, 107–116 (2004).

68. C. De Jonge, E. E. Kuramae, D. Radujković, J. T. Weedon, I. A. Janssens, F. Peterse, The influence of soil chemistry on branched tetraether lipids in mid-and high latitude soils: Implications for brGDGT-based paleothermometry. *Geochim. Cosmochim. Acta* **310**, 95–112 (2021).

69. C. De Jonge, D. Radujković, B. D. Sigurdsson, J. T. Weedon, I. Janssens, F. Peterse, Lipid biomarker temperature proxy responds to abrupt shift in the bacterial community composition in geothermally heated soils. *Org. Geochem.* **137**, 103897 (2019).

70. D. J. van Hinsbergen, L. V. De Groot, S. J. van Schaik, W. Spakman, P. K. Bijl, A. Sluijs, C. G. Langereis, H. Brinkhuis, A paleolatitude calculator for paleoclimate studies. *PLOS ONE* **10**, e0126946 (2015).

71. C. Diessel, R. Boyd, J. Wadsworth, D. Leckie, G. Chalmers, On balanced and unbalanced accommodation/peat accumulation ratios in the Cretaceous coals from Gates Formation,

Western Canada, and their sequence-stratigraphic significance. *Int. J. Coal Geol.* **43**, 143–186 (2003).

72. E. C. Hopmans, S. Schouten, J. S. Sinninghe Damsté, The effect of improved chromatography on GDGT-based palaeoproxies. *Org. Geochem.* **93**, 1–6 (2016).

73. S. Schouten, E. C. Hopmans, J. S. Sinninghe Damsté, The effect of maturity and depositional redox conditions on archaeal tetraether lipid palaeothermometry. *Org. Geochem.* **35**, 567–571 (2004).

74. J. W. H. Weijers, P. Steinmann, E. C. Hopmans, S. Schouten, J. S. Sinninghe Damsté, Bacterial tetraether membrane lipids in peat and coal: Testing the MBT–CBT temperature proxy for climate reconstruction. *Org. Geochem.* **42**, 477–486 (2011).

75. A. Sluijs, J. Frieling, G. N. Inglis, K. G. Nierop, F. Peterse, F. Sangiorgi, S. Schouten, Late Paleocene–early Eocene Arctic Ocean sea surface temperatures: Reassessing biomarker paleothermometry at Lomonosov Ridge. *Clim. Past* **16**, 2381–2400 (2020).

76. C. De Jonge, A. Stadnitskaia, E. C. Hopmans, G. Cherkashov, A. Fedotov, J. S. Sinninghe Damsté, In situ produced branched glycerol dialkyl glycerol tetraethers in suspended particulate matter from the Yenisei River, Eastern Siberia. *Geochim. Cosmochim. Acta* **125**, 476–491 (2014).

77. P. D. Hughes, G. Mallon, A. Brown, H. J. Essex, J. D. Stanford, S. Hotes, The impact of high tephra loading on late-Holocene carbon accumulation and vegetation succession in peatland communities. *Quat. Sci. Rev.* **67**, 160–175 (2013).

78. J. L. Ratcliffe, D. J. Lowe, L. A. Schipper, M. J. Gehrels, A. D. French, D. I. Campbell, Rapid carbon accumulation in a peatland following Late Holocene tephra deposition. *Quat. Sci. Rev.* **246**, 106505 (2020).

79. R. Payne, J. Blackford, Distal volcanic impacts on peatlands: Palaeoecological evidence from Alaska. *Quat. Sci. Rev.* **27**, 2012–2030 (2008).

80. S. S. Crowley, D. A. Dufek, R. W. Stanton, T. A. Ryer, The effects of volcanic ash disturbances on a peat: Forming environment: Environmental disruption and taphonomic consequences. *Palaios* **9**, 158–174 (1994).

81. S. S. Dirghangi, M. Pagani, M. T. Hren, B. J. Tipple, Distribution of glycerol dialkyl glycerol tetraethers in soils from two environmental transects in the USA. *Org. Geochem.* **59**, 49–60 (2013).

82. C. I. Blaga, G. J. Reichart, O. Heiri, J. S. Sinninghe Damsté, Tetraether membrane lipid distributions in water-column particulate matter and sediments: A study of 47 European lakes along a north–south transect. *J. Paleolimnol.* **41**, 523–540 (2009).

83. E. D. Crampton-Flood, J. E. Tierney, F. Peterse, F. M. Kirkels, J. S. Sinninghe Damsté, Global soil and peat branched GDGT compilation dataset. PANGAEA (2019); https://doi.org/10.1594/PANGAEA.907818.
